# Supplementary material for: Epigenetic age acceleration and clinical outcomes in gliomas
Source: PLoS One. 2020 Jul 21;15(7):e0236045. doi: 10.1371/journal.pone.0236045 (PMC7373289; doi:10.1371/journal.pone.0236045)
Supplement: S1 Fig — (DOCX) [file pone.0236045.s001.docx]

**S1 Figure**. Kaplan-Meier curves for patient overall survival in different clinical groups. (A) age group. (B) WHO grade. (C) Race. (D) Histology type.
