# Supplementary material for: Epigenetic age acceleration and clinical outcomes in gliomas
Source: PLoS One. 2020 Jul 21;15(7):e0236045. doi: 10.1371/journal.pone.0236045 (PMC7373289; doi:10.1371/journal.pone.0236045)
Supplement: S2 Fig — (DOCX) [file pone.0236045.s002.docx]

**S2 Figure.** Kaplan-Meier curves for patient overall survival between epigenetic age acceleration and epigenetic age deceleration in different tumor grades. (A) Grade II. (B) Grade III. (C) Grade IV.
