# Supplementary material for: Epigenetic age acceleration and clinical outcomes in gliomas
Source: PLoS One. 2020 Jul 21;15(7):e0236045. doi: 10.1371/journal.pone.0236045 (PMC7373289; doi:10.1371/journal.pone.0236045)
Supplement: S3 Fig — (DOCX) [file pone.0236045.s003.docx]

**S3 Figure**. Kaplan-Meier curves for patient overall survival between epigenetic age acceleration and epigenetic age deceleration in different histology subtypes. (A) Astrocytoma. (B) Glioblastoma. (C) Oligoastrocytoma. (D) Oligodendroglioma.
