# Supplementary material for: Epigenetic age acceleration and clinical outcomes in gliomas
Source: PLoS One. 2020 Jul 21;15(7):e0236045. doi: 10.1371/journal.pone.0236045 (PMC7373289; doi:10.1371/journal.pone.0236045)
Supplement: S1 Table — (DOCX) [file pone.0236045.s004.docx]

**S1 Table** Patient characteristics of original data from TCGA

|  | **LGG (n=516)** | | **GBM (n=140)** | |
| --- | --- | --- | --- | --- |
|  | **Count** | **Percentage (%)** | **Count** | **Percentage (%)** |
| **Age** |  |  |  |  |
| < 60 years | 452 | 87.6 | 65 | 46.4 |
| > 60 years | 62 | 12 | 73 | 52.1 |
| Unknown | 2 | 0.4 | 2 | 1.43 |
| **Gender** |  |  |  |  |
| Female | 230 | 44.6 | 58 | 41.4 |
| Male | 285 | 55.2 | 80 | 57.1 |
| Unknown | 1 | 0.2 | 2 | 1.43 |
| **Race** |  |  |  |  |
| Asian | 8 | 1.55 | 0 | 0 |
| Black | 21 | 4.07 | 0 | 0 |
| Native | 1 | 0.194 | 0 | 0 |
| White | 475 | 92.1 | 107 | 76.4 |
| Unknown | 11 | 2.13 | 33 | 23.6 |
| **Histology** |  |  |  |  |
| Astrocytoma | 169 | 32.8 | 0 | 0 |
| Oligoastrocytoma | 114 | 22.1 | 0 | 0 |
| Oligodendroglioma | 174 | 33.7 | 0 | 0 |
| Glioblastoma | 0 | 0 | 133 | 95 |
| Unknown | 59 | 11.4 | 7 | 5 |
| **Tumor grade** |  |  |  |  |
| G2 | 216 | 41.9 | 0 | 0 |
| G3 | 241 | 33.7 | 0 | 0 |
| G4 | 0 | 0 | 133 | 95 |
| Unknown | 59 | 11.4 | 7 | 5 |
| **Molecular subtype** |  |  |  |  |
| Classic-like | 23 | 4.46 | 52 | 37.14 |
| Codel | 173 | 33.5 | 0 | 0 |
| G-CIMP-high | 234 | 45.3 | 6 | 4.29 |
| G-CIMP-low | 12 | 2.33 | 0 | 0 |
| Mesenchymal-like | 45 | 8.72 | 56 | 40 |
| PA-like | 26 | 5.04 | 0 | 0 |
| Unknown | 3 | 0.581 | 26 | 18.58 |
