# Supplementary material for: Epigenetic age acceleration and clinical outcomes in gliomas
Source: PLoS One. 2020 Jul 21;15(7):e0236045. doi: 10.1371/journal.pone.0236045 (PMC7373289; doi:10.1371/journal.pone.0236045)
Supplement: S2 Table — (DOCX) [file pone.0236045.s005.docx]

**S2 Table** Patient characteristics of validation data

|  | **Patient (%)** | **Epigenetic age acceleration** | **p value ^a^** |
| --- | --- | --- | --- |
|  |  | **Mean (years)** |  |
| **Age** |  |  |  |
| < 60 years | 214 (93.4) | 0.38 | 0.583 |
| > 60 years | 15 (6.55) | -5.42 |  |
| **Gender** |  |  |  |
| Female | 89 (38.9) | -1.26 | 0.172 |
| Male | 128 (55.9) | -0.21 |  |
| Unknown | 12 (5.24) | 11.59 |  |
| **Histology** |  |  |  |
| Glioblastoma | 77 (33.6) | 1.16 | 0.331 |
| Oligoastrocytoma | 11 (4.8) | -11.1 |  |
| Oligodendroglioma | 21 (9.17) | 5.82 |  |
| Pediatric.Glioma | 59 (25.8) | -1.51 |  |
| Pilocytic astrocytoma | 61 (26.6) | 0 |  |
| **Tumor grade** |  |  |  |
| G1 | 61 (26.6) | 0 | 0.185 |
| G2 | 16 (6.99) | -8.68 |  |
| G3 | 16 (6.99) | 8.68 |  |
| G4 | 136 (59.4) | 0 |  |
| **Molecular subtype** |  |  |  |
| Codel | 18 (7.86) | 20.41 | **1.27E-05** |
| Classic-like | 24 (10.5) | 12.58 |  |
| G-CIMP-high | 17 (7.42) | -1.92 |  |
| G-CIMP-low | 8 (3.49) | 4.25 |  |
| Mesenchymal-like | 70 (30.6) | -3.22 |  |
| PA-like | 92 (40.2) | -4.84 |  |

^a^ Univariate analysis of the association of epigenetic age acceleration with clinical variable.

For 2-level variables, t-test was used, for more than 2-level variables, one-way ANOVA test

was used. Unknown data were not used in tests.
