# Supplementary material for: Epigenetic age acceleration and clinical outcomes in gliomas
Source: PLoS One. 2020 Jul 21;15(7):e0236045. doi: 10.1371/journal.pone.0236045 (PMC7373289; doi:10.1371/journal.pone.0236045)
Supplement: S3 Table — (DOCX) [file pone.0236045.s006.docx]

**S3 Table** The associations of epigenetic age acceleration with clinical variables in validation data ^a^

|  | **Epigenetic age  acceleration (years)** | **95% CI  (Lower)** | **95% CI  (Higher)** | **P value** |
| --- | --- | --- | --- | --- |
| **Molecular subtype (Codel as Ref.)** | | | | |
| Classic-like | -16.49 | -30.62 | -2.37 | 2.23E-02 * |
| G-CIMP-high | -26.94 | -40.72 | -13.16 | 1.54E-04 *** |
| G-CIMP-low | -28.26 | -46.52 | -10.01 | 2.56E-03 ** |
| Mesenchymal-like | -34.28 | -46.49 | -22.06 | 9.13E-08 *** |
| PA-like | -44.43 | -57.65 | -31.22 | 2.63E-10 *** |
| **Age (<=60 as Ref.)** | | | | |
| > 60 years | -16.74 | -28.26 | -5.23 | 4.56E-03 *** |
| **Tumor grade (G2 as Ref.)** | | | | |
| G1 | 34.28 | 20.08 | 48.47 | 3.56E-06 *** |
| G3 | 23.49 | 9.06 | 37.92 | 1.54E-03 ** |
| G4 | 22.53 | 10.51 | 34.55 | 2.78E-04 *** |

^a^ Multivariate linear regression was used to study the association of epigenetic age acceleration with SDM, adjusted by age and tumor grade. * p < 0.05, ** p < 0.01, *** p < 0.001
