# Supplementary material for: Epigenetic age acceleration and clinical outcomes in gliomas
Source: PLoS One. 2020 Jul 21;15(7):e0236045. doi: 10.1371/journal.pone.0236045 (PMC7373289; doi:10.1371/journal.pone.0236045)
Supplement: S4 Table — (DOCX) [file pone.0236045.s007.docx]

**S4 Table** Overall survival of gliomas patients in validation dataset using multivariate analysis

|  | **HR** | **95% CI (Lower)** | **95% CI (Upper)** | **P value ^a^** |
| --- | --- | --- | --- | --- |
| **Epigenetic Age acceleration** | 0.998 | 0.876 | 1.137 | 0.974 |
| **Molecular subtype (Codel as Ref.)** | | | | |
| Classic-like | 20.850 | 4.338 | 100.206 | **1.49E-04** |
| G-CIMP-high | 2.719 | 0.564 | 13.102 | 0.213 |
| Mesenchymal-like | 13.695 | 3.285 | 57.091 | **3.27E-04** |
| PA-like | 14.481 | 2.912 | 72.011 | **1.09E-03** |
| **Age (<=60 as Ref.)** | | | | |
| > 60 years | 1.498 | 0.336 | 6.681 | 0.596 |
| **Tumor grade (G2 as Ref.)** | | | | |
| G3 | 3.783 | 0.979 | 14.620 | 0.0537 |
| G4 | 1.498 | 0.455 | 4.928 | 0.506 |

^a^ Cox proportional hazards regression was used for multivariate survival analysis to assess the association of patient characteristic with overall survival
