# Supplementary material for: Epigenetic age acceleration and clinical outcomes in gliomas
Source: PLoS One. 2020 Jul 21;15(7):e0236045. doi: 10.1371/journal.pone.0236045 (PMC7373289; doi:10.1371/journal.pone.0236045)
Supplement: S5 Table — (DOCX) [file pone.0236045.s008.docx]

**S5 Table** The association of age acceleration with patient overall survival in stratified analyses ^a^

|  | **Alive** | **Dead** | **Death rate  (%)** | **HR** | **95% CI  (Lower)** | **95% CI  (Upper)** | **P value** |
| --- | --- | --- | --- | --- | --- | --- | --- |
| **Age** |  |  |  |  |  |  |  |
| *<= 60 years* | *101* | *68* | *40.2* | *0.930* | *0.842* | *1.027* | *0.152* |
| > 60 years | 4 | 3 | 42.9 | 1.007 | 0.729 | 1.391 | 0.968 |
| **Tumor grade** |  |  |  |  |  |  |  |
| *G2* | *13* | *4* | *23.5* | *0.772* | *0.466* | *1.281* | *0.317* |
| *G3* | *11* | *9* | *45* | *0.887* | *0.707* | *1.112* | *0.299* |
| *G4* | *81* | *58* | *41.7* | *0.970* | *0.853* | *1.103* | *0.641* |
| **Molecular subtype** |  |  |  |  |  |  |  |
| Classic-like | 11 | 9 | 45 | 0.998 | 0.876 | 1.137 | 0.974 |
| Codel | 14 | 4 | 22.2 | 1.707 | 0.739 | 3.943 | 0.211 |
| G-CIMP-high | 10 | 4 | 28.6 | 1.707 | 0.739 | 3.943 | 0.211 |
| *Mesenchymal-like* | *47* | *35* | *42.7* | *0.846* | *0.696* | *1.029* | *0.094* |
| PA-like | 23 | 19 | 45.2 | 1.085 | 0.832 | 1.413 | 0.547 |

^a^ Cox proportional hazards regression was used for univariate survival analysis to assess the association of patient characteristic with overall survival in each group. Italicized groups show the trend of positive association of epigenetic age acceleration with patient overall survival
